# Zika Virus Inhibits IFN-α Response by Human Plasmacytoid Dendritic Cells and Induces NS1-Dependent Triggering of CD303 (BDCA-2) Signaling

**DOI:** 10.3389/fimmu.2020.582061

**Published:** 2020-10-28

**Authors:** Sandra Bos, Béatrice Poirier-Beaudouin, Valérie Seffer, Maria Manich, Cartini Mardi, Philippe Desprès, Gilles Gadea, Marie-Lise Gougeon

**Affiliations:** ^1^ Institut Pasteur, Innate Immunity and Viruses Unit, Global Health Department, Paris, France; ^2^ Université de la Réunion, INSERM U1187, CNRS UMR 9192, IRD UMR 249, Unité Mixte Processus Infectieux en Milieu Insulaire Tropical, Plateforme Technologique CYROI, La Réunion, France; ^3^ Institut Pasteur, Biological Image Analysis Unit, Cell Biology and Infection Department, Paris, France

**Keywords:** Zika virus (ZIKV), plasmacytoid dendritic cells (pDC), IFN—interferon, CD303, non-structural protein 1 (NS1)

## Abstract

Zika virus (ZIKV) dramatically emerged in French Polynesia and subsequently in the Americas where it has been associated with severe neurological complications in adults and newborns, respectively. Although plasmacytoid dendritic cells (pDCs) are a key sensor of viral infection and are critical for initiating an antiviral response, little is known about the impact of ZIKV infection on pDCs. Here, we investigated the susceptibility of human pDCs to infection with multiple strains of ZIKV and further investigated the impact of infection on pDCs functions. We observed that pDCs were refractory to cell-free ZIKV virions but were effectively infected when co-cultured with ZIKV-infected cells. However, exposure of pDCs to ZIKV-infected cells resulted in limited maturation/activation with significant down regulation of CD303 expression, a severe impairment of inflammatory cytokine production, and an inability to mount an IFN-α response. We show that ZIKV developed a strategy to inhibit the IFN-α response in primary human pDCs likely mediated through NS1-dependent CD303 signaling, thus suggesting a new mechanism of immune evasion.

## Introduction

Zika virus (ZIKV) is a single stranded RNA virus belonging to flavivirus genus of the Flaviviridae family, closely related to the mosquito-borne flaviviruses dengue virus (DENV), West Nile virus (WNV), yellow fever virus (YFV), and Japanese encephalitis virus (JEV) (1). Until the first outbreaks in South Pacific and then the epidemic in Americas in 2015, ZIKV has long been thought to only cause mild infections in humans with low viral pathogenicity (2). However, ZIKV caused significant neuropathology in humans during the recent outbreaks, such as Guillain-Barre Syndrome (3), and fetal microcephaly, termed Zika congenital syndrome, and other birth defects associated to neurological disorders (4). Potential factors contributing to increased pathogenicity of the recently emerged strains can relate to viral genetic diversity, cell tropism and host cell damages, magnitude of viral replication and virus persistence, and the balance between the host antiviral response and immune evasion of the virus (5).

The innate immune system is the first line of host defense against viruses, and type I Interferons (IFN-I) are induced as a key component of this response to upregulate antiviral genes, known as IFN-stimulated genes (ISGs). IFNs and ISGs promote a cell-autonomous antiviral state but they also trigger an inflammatory response and adaptive immunity (6). The critical role of the IFN-I response in controlling ZIKV infection is supported by the fact that Ifnar1−/− mice lacking IFN-α/β receptor and Irf3−/− Irf5−/− Irf7−/− triple knockout mice, which don’t produce IFN-α/ß, are highly vulnerable to ZIKV and more likely to develop signs of neurological disease associated with high viral load in the brain and to die within 1 week of infection compared to immunocompetent WT mice (7). IFN-induced protection from ZIKV is also suggested by the finding that the injection in WT mice of an antibody that targets the IFNAR-1 subunit of the IFN-α/β receptor leads to 40%–100% mortality when these mice are exposed to ZIKV, and also acute encephalitis characterized by neuronal death, microgliosis, and inflammatory cell infiltrates (8).

Although most cells can produce IFN-I, plasmacytoid dendritic cells (pDCs) are specialized and the most potent producers in response to most viruses (9, 10). pDCs are a rare cell type in the peripheral blood, their response is rapid and triggered by the endosomal sensors Toll-like receptor (TLR) 7 and TLR9, which recognize viral nucleic acids (RNA and DNA, respectively) (11, 12). The interactions of pDCs with arboviruses have mostly been studied in response to DENV, YFV, and Chikungunya virus (CHIKV). pDCs were found to poorly support DENV replication. However, the sensing of DENV-infected cells triggers their maturation and expression of costimulatory molecules, and induces a TLR-7 dependent IFN-α response and secretion of inflammatory cytokines, including TNF-α (13, 14). This cell-to-cell sensing involves the transmission of viral components that are clustered at the interface between pDCs and infected cells. It was also reported that non-infectious DENV immature particles are potent inducers of IFN-α response in pDCs (14). Similarly, cells producing immature YFV particles were more potent stimulators of pDCs IFN-α response than cells releasing mature virions (15). Furthermore, mice genetically engineered so that the production of IFN was restricted to pDCs resisted to disease induced by DENV or CHIKV infection, showing that pDCs have an outsize role as first responders and as coordinators of the immune response (9). Altogether, these studies suggest that arboviruses are able to trigger an antiviral state in pDCs, thus contributing to the control of the infection at an early stage.

Although pDC sensing of infected cells and subsequent triggering of innate antiviral response has been demonstrated for DENV, YFV, and CHIKV, the role of pDCs in the early events following exposure to ZIKV is currently unknown. Recent work has shown that ZIKV Inhibits IFN-I protein translation along with limited secretion of inflammatory cytokines in human monocyte-derived DCs (mDCs), another DC subset, despite strong induction at the RNA transcript level and up-regulation of other host antiviral proteins. This was associated with the inhibition of type I IFN receptor signaling through blockade of STAT1 and STAT2 phosphorylation (16). Thus, ZIKV can subvert mDC immunogenicity during infection in part through evasion of type I IFN responses. Nevertheless, the impact of ZIKV on IFN-I response triggering in pDCs and the induction of their maturation and cytokine response remains unknown.

In order to address this question, we studied the antiviral response of primary human pDCs to two ZIKV strains i.e. the historical MR766 strain of African lineage and the epidemic contemporary strain BeH819015 of Asian lineage isolated in 2015 from human serum specimen in Brazil. Herein, we report that pDCs exposure to ZIKV-infected cells resulted in a very limited maturation/activation of pDCs with significant down regulation of CD303 expression and a lack of IFN-α response. CD303 triggering in pDCs has been shown to exert a negative regulation of the NF-kB pathway and to inhibit IFN-I genes *via* a BCR-like signaling involving tyrosine phosphorylation of SYK (17). Here we show that ZIKV developed a strategy to inhibit the IFN-α response in primary human pDCs and induces CD303 signaling and SYK phosphorylation in a NS1-dependent manner.

## Materials and Methods

### Cells and Viruses

Vero cells (ATCC, CCL-81) and IMR32 cells (ATCC, CCL-127) were cultured at 37°C in a humidified 5% CO2 chamber in complete culture medium composed of MEM supplemented with 5% or 10% FBS respectively, 1% penicillin-streptomycin, 2 mmol L−1 l-Glutamine, and 1 mmol L−1 sodium pyruvate (PAN Biotech). The culture medium of IMR32 cells was enriched with 5% non-essential amino acids (PAN Biotech). ZIKV BR15 and MR766 stocks were prepared on Vero cells infected with molecular clones of BeH819015 strain (GenBank access KU365778), and historical MR766 Uganda 47-NIID strain (Genbank access LC002520) respectively; both molecular clones were previously described (18). Virus-free supernatant from Vero cells (SN) produced upon the same culture condition, and cell batch, were collected along with ZIKV stocks to be used as a control. YF-17D stock was prepared on Vero cells inoculated with the YFV vaccine strain (YF-17D-204 STAMARIL, Sanofi Pasteur, Lyon) provided by the Institut Pasteur Medical Center. Viral titers were determined by a standard plaque-forming assay on Vero cells as previously described (18). Viral stock used in the following experiments were collected at passage 2, with infectious titer at 2.106 PFU.ml-1 for ZIKV BR15 and YF-17D and 2.107 PFU.ml-1 for ZIKV MR766.

### Isolation and Preparation of pDCs

Peripheral Blood Mononuclear Cells (PBMCs) were separated from the blood of healthy adult donors on a Ficoll-Hypaque density gradient. Blood was obtained through the EFS (Etablissement Français du Sang) in the setting of EFS-Institut Pasteur Convention. pDCs were isolated from fresh PBMCs as previously reported (19) using the Human Plasmacytoid DC Negative Isolation Kit (EASYstep, StemCell Technologies). The enriched cells were assessed for more than 95% purity using the following antibodies: CD123–APC (clone AC145), CD3-V500 (clone UCHT1) purchased from BD Horizon and CD303–PE (clone REA693) purchased from MACS. pDCs were cultured in complete medium, composed of RPMI 1640 (Invitrogen, Gaithersburg, MD, USA), 10% FBS and 1% penicillin-streptomycin, at 37°C in a humidified 5% CO^2^ chamber according to protocol. When indicated, pDCs were stimulated overnight with CpG ODN 2006 (InvivoGen, USA) at 3 ug/ml.

### Infection of pDCs

Freshly isolated pDCs were incubated with ZIKV MR766 or BR15 at a multiplicity of infection (MOI) of 1 and 5 (referred to as low and high input respectively), with YF-17D at MOI of 1, or with virus-free supernatant (SN) in an equal volume to ZIKV BR15 maximum input. After 2 h of incubation, culture medium was changed and pDCs were cultured at 37°C in a humidified 5% CO_2_ chamber in complete medium for 24 h.

### Co-Culture Experiments

Vero and IMR32 cells were infected at MOI of 1 and 4 respectively. Twelve hours post-infection the culture medium was removed and replaced with complete RPMI medium containing 5 × 10^4^ pDCs. Cells were co-cultured at 37°C in a humidified 5% CO2 chamber.

### Flow Cytometry Analyses

The phenotype of pDCs was assessed with the following primary mAbs (BD Horizon): CCR7-FITC (clone 3D12), CD83-PE-Cy7 (clone HB15e), CD86-PE-Cy5 (clone 233), HLA-DR-APC-H7 (clone L243), and CD123-PerCP-Vio 700 (clone AC145). CD303-PE (clone REA693) was purchase from MACS. Cells were fixed with 4% PFA for 20 min, stained for 30 min at 4°C and washed before being subjected to FACS analysis. ZIKV infectivity was assessed with the mouse anti-pan flavivirus envelope protein mAb 4G2 (RD Biotech). A ZIKV-specific polyclonal antibody targeting the envelope protein and the non-structural protein 1 (20) or the mouse anti-dsRNA IgG2a mAb J2 (Scicons) as indicated. Cells were fixed with 4% PFA for 20 min at room temperature (RT) and permeabilized with 0.1% Triton X-100 in PBS for 4 min at RT. Fixed cells were stained overnight at 4°C using 4G2 or J2 (1:500) in PBS-BSA. Then, cells were stained 20 min at RT with secondary antibody donkey anti-mouse Alexa Fluor 488 IgG (1:1000, Invitrogen) or donkey anti-mouse Cy3 (1:500, Jackson immunoResearch) in PBS-BSA, and washed before being subjected to FACS analysis. At least, 5,000 events were acquired using Cyan cytometer (Beckman Coulter). Stained cells were analyzed using FlowJo software (Tree Star, Inc., Ashaland, OR). pDCs survival was assessed using the 7-AAD assay as previously described (21).

### Cell Viability Assay

Cell viability was determined using the CytoTox 96^®^ Non-Radioactive Cytotoxicity Assay (Promega) according to the manufacturer’s recommendations. This assay is based on LDH release quantification as indicator of cell death.

### Cytokines and Chemokines Measurement

Chemokines and cytokines were measured by Luminex (Human XL cytokine Premixed Magnetic Luminex Performance Assay Kit (R&D Systems, bio-techne) according to the manufacturer’s instructions. In brief, 50 µl of standard or supernatant inactivated with 1% NP40 for 10 min at 4°C were incubated with antibody-linked beads for 2 h. Then samples were washed three times with wash solution, and incubated for 1 h with biotinylated secondary antibodies. A final incubation of 30 min with streptavidin-PE preceded the acquisition on the Bioplex 200 (Biorad). At least 100 events were acquired for each analyte. Values below the standard curves were replaced by the lowest values of the concentrations measured.

### Immunofluorescence and Confocal Analysis

Freshly isolated pDCs were stained with the Cell Tracker Deep Red Dye (Thermo Fisher Scientific) for 30 min at 37°C. Labeled pDCs were co-cultured for 24 h with previously seeded and infected Vero cells in glass coverslips. Cells were fixed with 37°C-prewarmed PFA to a final concentration of 4% (v/v) for 20 min at room temperature (RT) and permeabilized with 0.1% Triton X-100 in PBS for 4 min at RT. Fixed cells were stained overnight at 4°C using a polyclonal anti-ZIKV antibody provided by P. Desprès (1:1000 dilution) in PBS-BSA. Then, cells were stained 20 min at RT with the secondary antibody donkey anti-mouse Alexa Fluor 488 IgG (Molecular Probes, 1:1000 dilution) in PBS-BSA. Nuclei were stained with NucBlue (Thermo Fisher Scientifc) for 20 min at RT. Lastly, coverslips were washed with BSA-free PBS and mounted with ProLong Glass antifade reagent (Molecular Probes, USA). Z-sections across cells at step size increments of 0.5-μm increments were acquired with a Zeiss LSM 700 laser scanning confocal microscope (LSM 700, Zeiss, Germany) equipped with a X63 objectives, numerical aperture NA=1.4. Images were analyzed with the Icy software (icy.bioimageanalysis.org) (22).

### Phosflow Assay

Phosflow was performed to measure the phosphorylation state of intracellular SYK following stimulation. Purified pDCs were plated at 8×10^4^ cells per well and stimulated with ZIKV BR15, virus-free supernatant (SN), PBS-BSA 2% (mock), 1 µg/ml anti-BDCA-2 mAb (Miltenyi Biotec), or 2,5 µg/ml pure ZIKV-NS1 (R&D System, accession number AHZ13508) in PBS-BSA 2% for 30 min. Stimulations were performed in a volume of 120 µl (equivalent of BR15 MOI of 3) to allow experimentation in 96-well plates. Cells were fixed in a volume-to-volume prewarmed BD Phosflow Fix Buffer I (BD Biosciences) and then permeabilized with BD Phosflow Perm buffer III (BD Biosciences) according to manufacturer instructions. Intracellular phosphorylated SYK was stained with p-SYK (Y525/526) and PE-anti-Rabbit IgG antibodies (Cell Signaling Technologies). Cells were washed before being subjected to FACS analysis. Stained cells were analyzed using the FlowJo software (Tree Star, inc, Ashaland, OR).

### Statistical Analysis

The data are presented as arithmetic mean ± SD. Comparisons between different treatments have been analyzed by a one-way or two-way ANOVA tests as appropriate. Values of p < 0.05 were considered statistically significant for a post-hoc Tukey’s test. Statistical analysis was performed using GraphPad Prism version 8 (GraphPad software, San Diego, CA).

## Results

### Limited pDC Maturation in Response to ZIKV Exposure

pDCs were purified from freshly isolated PBMC from healthy donors and subjected to flow cytometry analysis. *Ex-vivo* sorted pDCs were all CD123^+^CD303^+^HLA-DR^+^, with low expression of CD83, CD86, and CCR7, thus exhibiting the phenotype of immature pDCs (19). pDCs phenotype was then analyzed 24 h after exposure to ZIKV BR15 or MR766 (MOI of 1 and 5). Freshly isolated pDCs, pDCs incubated in complete medium (Mock) or with the supernatant (SN) of uninfected Vero cells (see *Materials and Methods*), and pDCs stimulated with the TLR9 agonist CpG oligodeoxynucleotides (CpG ODN) 2006 were used as controls for the analysis of FSC/SSC parameters and expression of activation markers.

**Figure 1:**
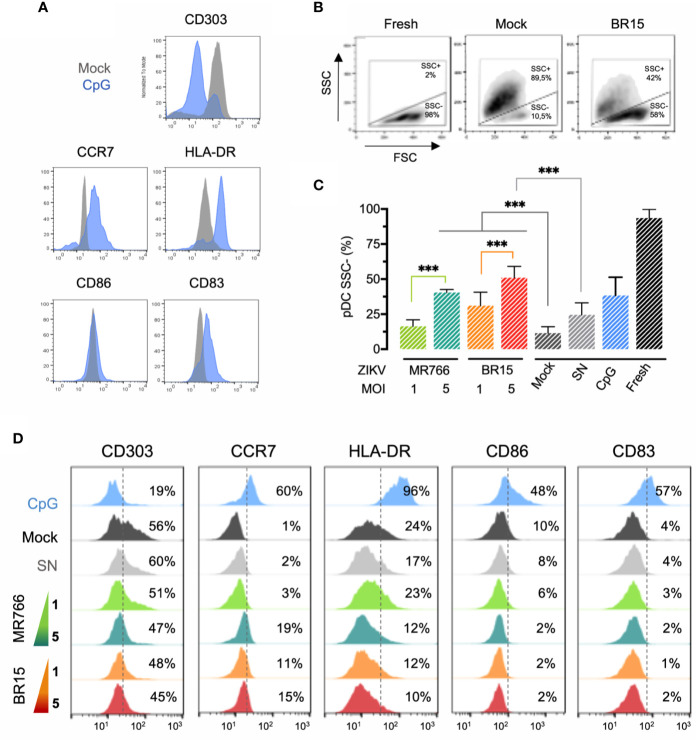
Impact of ZIKV on pDC maturation **(A)** Phenotypic characterization of mature pDCs. Sorted pDCs were either incubated with medium (Mock) or stimulated with ODN 2006 for 24 h and the expression of the indicated markers was analyzed. **(B)** Repartition of pDCs within two sub-populations according to FSC/SSC parameters. Shown are representative density plots of pDCs freshly isolated, incubated with medium, or exposed to ZIKV BR15 at MOI of 5. **(C)** Proportion of freshly isolated pDCs or pDCs cultured for 24 h clustered within the SSC^low^ populations. Cultured cells were either incubated with medium (mock), stimulated with ODN 2006, or exposed to different MOI of indicated ZIKV strain or to virus-free supernatant (SN). The error bars represent the standard deviations of at least three experiments conducted with primary cells from distinct donors, except for ZIKVs MOI of 5 (two donors in duplicate). **(D)** Phenotypic characterization of pDCs cultured for 24 h. pDCs were either incubated with medium (mock), stimulated with ODN 2216, or exposed to different MOI of indicated ZIKV strain or to virus-free supernatant (SN), and the expression of the indicated maturation markers was analyzed. The dotted line represents the positive expression limit of the specified marker and the percentage of cells expressing it is indicated. Results shown in panels **(A, B, D)** are representative of at least three experiments conducted with primary cells from distinct donors, except for ZIKVs MOI of 5 (two donors in duplicate). ***p < 0.0001.

TLR9-dependent stimulation of pDCs with CpG ODN 2006 induced their maturation, as evidenced by the increased expression of CCR7, HLA-DR and CD83 markers, and decreased expression of CD303, also a hallmark of pDC maturation (23) **(** **Figure 1A** **)**. pDCs characterization was then performed following 24 h exposure to ZIKV BR15 or MR766. **Figure 1B** shows FSC/SSC characteristics of either freshly isolated pDCs, or pDCs incubated in complete medium (Mock) or with ZIKV BR15 at MOI of 5. While freshly isolated immature pDCs were in majority a SSC^low^ population, as expected for living cells, pDCs incubated overnight in complete medium (Mock) were SSC^high^, indicating that they were dying because of the lack of survival signals, such as IL-3 (24). In contrast, pDCs exposed to ZIKV displayed to a large extent the features of living cells, with more than 50% of SSC^low^ cells upon exposure to BR15 at MOI of 5 (**Figure 1B**). The frequency of SSC^low^ pDCs increased in a viral dose-dependent manner, suggesting that ZIKV might have the ability to maintain pDC viability regardless of viral strain. A significant survival effect of ZIKV was detected for both strains at MOI of 5 compared to Mock condition, and CpG ODN 2006 had a similar effect (**Figure 1C**, **Supplemental Table 1**). The survival effect of both strains of ZIKV was confirmed using 7-amino actinomycin D (7-AAD) (**Figure S1**). Thus, sensing of ZIKV by pDCs was associated with their survival.

Surprisingly, this sensing was associated with a limited degree of pDC maturation (**Figure 1D**). Among the maturation markers tested, only CCR7, a chemokine receptor that promotes pDCs homing to lymph nodes (25) was upregulated in pDCs exposed to MR766 and BR15 at MOI of 5 (19% and 15% of pDCs expressed CCR7 compared to 1% in mock cultures. In contrast, the maturation markers HLA-DR, CD86, or CD83 were not upregulated in pDCs exposed to both strains of ZIKV, while they were highly induced by the TLR9 agonist CpG ODN 2006, thus evidencing pDC maturation (**Figure 1D**). Interestingly, a down-regulation of CD303 expression on the surface of pDCs was detected upon exposure to both strains of ZIKV compared to Mock condition **(** **Figure 1D**). This observation supports the hypothesis that the maturation defect of pDCs exposed to ZIKV is induced by CD303 triggering as antibody-mediated CD303 signaling in pDCs was shown to inhibit both their activation and antigen presentation (26).

### Susceptibility of pDCs to ZIKV Infection

**Figure 2:**
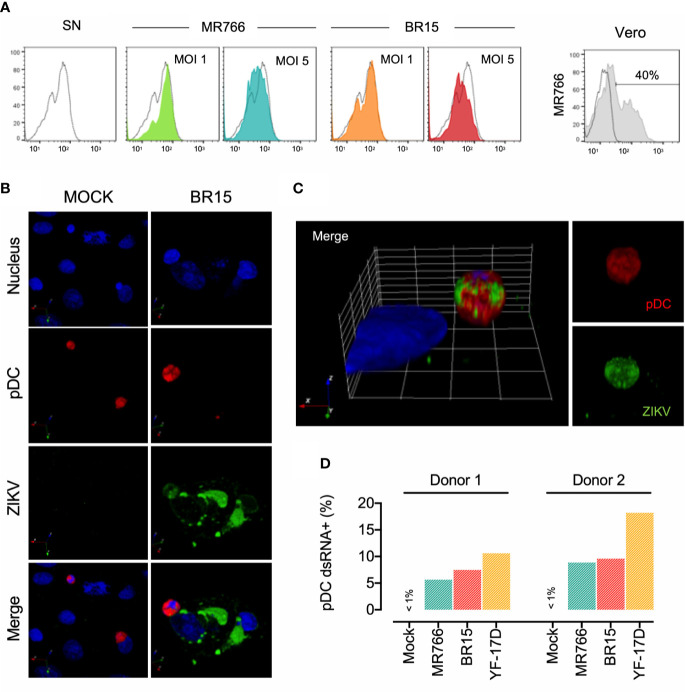
Susceptibility of pDC to ZIKV infection **(A)** ZIKV envelope protein expression in pDCs after 24 h of exposure to the virus. Histograms show 4G2 staining of pDCs exposed to virus-free supernatant (SN) as negative control, ZIKV MR766 or BR15 at MOI of 1 or 5 (colored histograms) and infected Vero cells (grey histogram). In this last histogram, dotted line corresponds to mock-infected Vero cells. The percentage of Vero cells expressing 4G2 is indicated. **(B)** Confocal microscopy analysis of pDCs exposed 24 h to virus-free supernatant (SN) or Vero cells infected with ZIKV BR15 at a MOI of 1. Cell nucleus was stained with NucBlue, pDCs were stained with the cell tracker Deep Red before their coculture with Vero cells, and ZIKV was detected intracellularly with the mouse polyclonal antibody (20), which recognizes Env and NS1 proteins. **(C)** 3D reconstitution of confocal microscopy acquisition of ZIKV-BR15 infected pDC. pDCs were labelled in red, the nucleus in blue, and ZIKV in green. Images are representative of three independent experiments conducted with primary cells from three distinct donors. **(D)** Frequency of pDCs infected by ZIKV. pDCs were cocultured 24 h with Vero cells uninfected (mock) or infected with ZIKV MR766 or BR15, or with YF-17D. The frequency of infected pDCs was determined following the intracellular staining with J2 anti-dsRNA antibody. Experiments performed with sorted pDCs from two distinct donors are shown.

We first evaluated the susceptibility of freshly isolated pDCs to ZIKV infection. Cells were exposed to low (MOI of 1) or high (MOI of 5) input of MR766 and BR15 viruses and the percentage of ZIKV-infected pDC was determined at 24 h p.i. by FACS analysis using the anti-*pan* flavivirus E protein mAb 4G2. The highly susceptible Vero cells were used as a positive control of ZIKV infection. As shown in **Figure 2A**, while 40% of infected Vero cells were stained with 4G2, no staining was detected in pDCs exposed to infectious cell-free virions MR766 or BR15 regardless the MOI used. Such a result confirms a previous report showing that pDCs are globally refractory cells to endocytic enveloped viruses due to the constitutive expression of RAB15 which alters the fusogenic capacity of the virion (27). In an effort to better understand the inherent resistance of pDCs to ZIKV infection, we performed co-culture experiments with ZIKV-infected Vero cells and pDCs. Vero cells are considered as a suitable model of feeder cells due to the lack of IFNα/β genes and the very low expression of maturation marker induced by their SN on fresh pDCs, as shown in **Figure 1D**. Viral protein expression among pDCs in close contact to ZIKV-infected Vero cells was first analyzed by confocal microscopy imaging on deep-red-labelled pDCs cocultured 24 h with infected Vero cells using a polyclonal ZIKV-specific antibody targeting the E and the non-structural protein 1 (NS1) (20). **Figure 2B** shows two pDCs (red staining) standing in close proximity to ZIKV-infected-Vero cell (nucleus blue staining) and expressing ZIKV proteins (green staining). Both pDC and Vero cell appeared as positive for ZIKV. As illustrated by the 3D reconstitution (**Figure 2C**), massive intracytoplasmic staining of ZIKV was observed in cocultured pDCs, highlighting their potency to carry ZIKV proteins once in contact to Vero cells in which virus is replicating. These results were extended by FACS analysis (**Figure 2D**). Intracellular staining with anti-dsRNA J2 mAb revealed that 5%–10% of pDCs were dsRNA+ after 24 h of co-culture with ZIKV-infected Vero cells, whatever the viral strain tested. As a positive control (15), live-attenuated YFV vaccine strain YF-17D induced 10%–15% of dsRNA^+^ pDCs under the same experimental conditions (**Figure 2D**). Taken together, these results strongly argue for a cell-to-cell transmission of ZIKV towards pDCs which are resistant to direct infection.

### Lack of IFN-α Response by pDCs Exposed to ZIKV-Infected Epithelial Cells

**Figure 3:**
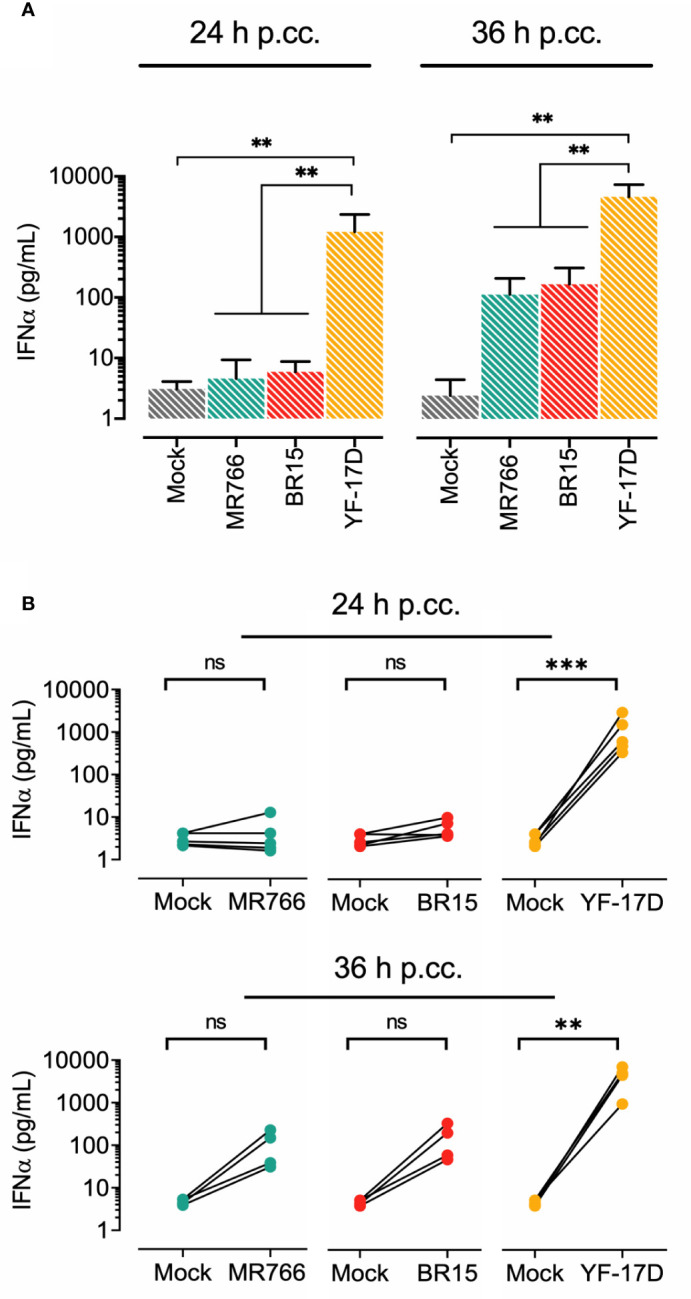
Lack of IFN-α production by pDCs exposed to ZIKV-infected epithelial cells **(A)** Quantification of IFN-α in the supernatant of pDCs co-cultured with Vero cells. Feeder cells were left either uninfected (mock) or infected for 12 h with the indicated flavivirus strain at MOI of 1 prior co-culture with pDCs for a further 24 to 36 h. Shown are mean/SD of results obtained upon 24 and 36 h of coculture from and four distinct donors respectively **(B)** Comparison of IFN-α production in pDCs co-cultured with indicated flavivirus strain at the two time points for each donor. **p < 0.001, ***p < 0.0001, ns, not significant.

It is known that pDCs are sentinel immune cells specialized in the rapid and massive production of IFN-α triggered by viral sensing, an innate response of central importance to protect the host against viral infections. Using a Luminex assay for cytokine quantification, we analyzed the amount of IFN-α produced by pDCs after 24 h of exposure to ZIKV. While CpG ODN 2216 triggered a strong IFN-α response, as expected, pDCs incubated with cell-free ZIKV failed to produce IFN-α regardless the viral strain tested (**Figure S2**). In agreement with the data reported by Sinigaglia et al. (15), the same outcome was observed upon YF-17D exposure, whereas an abundant amount of IFN-α (>1000 pg.ml-1) was secreted by pDCs co-cultured 24 h with YF-17D-infected Vero cells (**Figure 3A** **)**. In contrast, pDCs failed to produce IFN-α (<10 pg. ml-1) when exposed to MR766- or BR15- infected Vero cells (**Figure 3A**). At 36 h of co-culture, only a very low IFN-α response (about 100 pg.ml-1) was detected in pDCs exposed to ZIKV-infected cells, independently of the strain used, while a strong response leading to the secretion of more than 3000 pg.ml-1 of IFN-α was elicited in pDCs co-cultured with YF-17D-infected cells. The lack of IFN-α production in response to infection with BR15 or MR766 was highly reproductible for all donors (**Figure 3B** **)**. Feeder cells conditions were monitored at several key steps of the co-culture experiments in order to evaluate viral growth and cytotoxicity. Data in **Figure S3** show that Vero cells in monoculture and Vero cells co-cultured with pDCs were efficiently infected by MR766 and BR15 strains and allowed a significant release of virions with no to low cell death at 24 h p.i. and 48 h p.i. respectively. Therefore, the lack of IFN-α response in pDCs is not due to a defective viral production in cocultures.

### Lack of IFN-α Response by pDCs Exposed to ZIKV-Infected Human Neuroblastoma Cells

**Figure 4:**
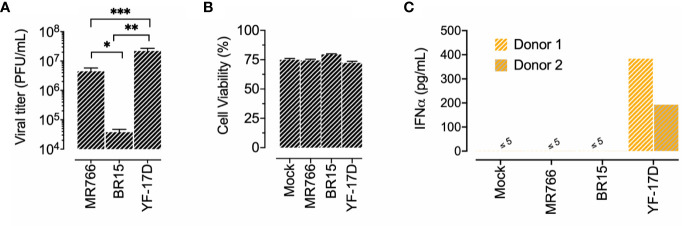
Lack of IFN-α production by pDCs exposed to ZIKV-infected neuroblastoma cells. IMR-32 cells were left uninfected (mock) or infected with the indicated flavivirus strain at MOI of 4. IMR-32 cells were infected for 12 h before their co-culture with pDCs for a further 24 h. **(A)**: Quantification of viral progeny production. The release of infectious virus in the supernatant of infected cells was titrated on Vero cells. **(B)** Analysis of cell viability was assessed 36 h post-IMR32 cell infection by LDH release measurement. Cell viability is expressed as percentage relative to maximum LDH release. Data in panels **(A, B)** are expressed as mean/SD of two experiments performed in duplicate. **(C)** Quantification of IFNα in the supernatant of pDCs co-cultured 24 h with IMR32 cells infected with the indicated flaviviruses. The results shown were obtained from two distinct donors. *p < 0.05; **p < 0.001; ***p < 0.0001.

Given the neurotropism of ZIKV and the capacity of pDCs to be recruited in CNS during flavivirus infection, as reported for WNV (28), we studied the interaction of freshly isolated pDCs with neuroblastoma cells IMR-32 infected by ZIKV. To this end, IMR-32 cells were infected with ZIKV strain BR15 or MR766 and co-cultured with pDCs under the same conditions as those described above for Vero cells. YF-17D served as a positive control. **Figure 4** shows viral progeny production and cell viability at 36 h post-infection i.e. at the end of the 24 h of co-culture experiment. We observed that efficacy of ZIKV replication in IMR-32 was greater with the viral strain MR766 when compared to BR15 (**Figure 4A**). However, no marked change in cell viability was detected at 36 h p.i. (**Figure 4B**). pDC IFN-α response following exposure to ZIKV-infected neuronal cells was then assessed. **Figure 4C** shows that while pDCs exposed to YF-17D-infected IMR-32 cells mounted a vigorous IFN-α response, none of the two strains of ZIKV triggered the secretion of IFN-α in pDCs. Therefore, defective IFN-α pDCs response appeared to be specific to ZIKV, and occurred independently of the type of feeder cells used, whether epithelial or neuronal, simian or human.

### ZIKV Does Not Trigger an Inflammatory Response in pDCs

**Figure 5:**
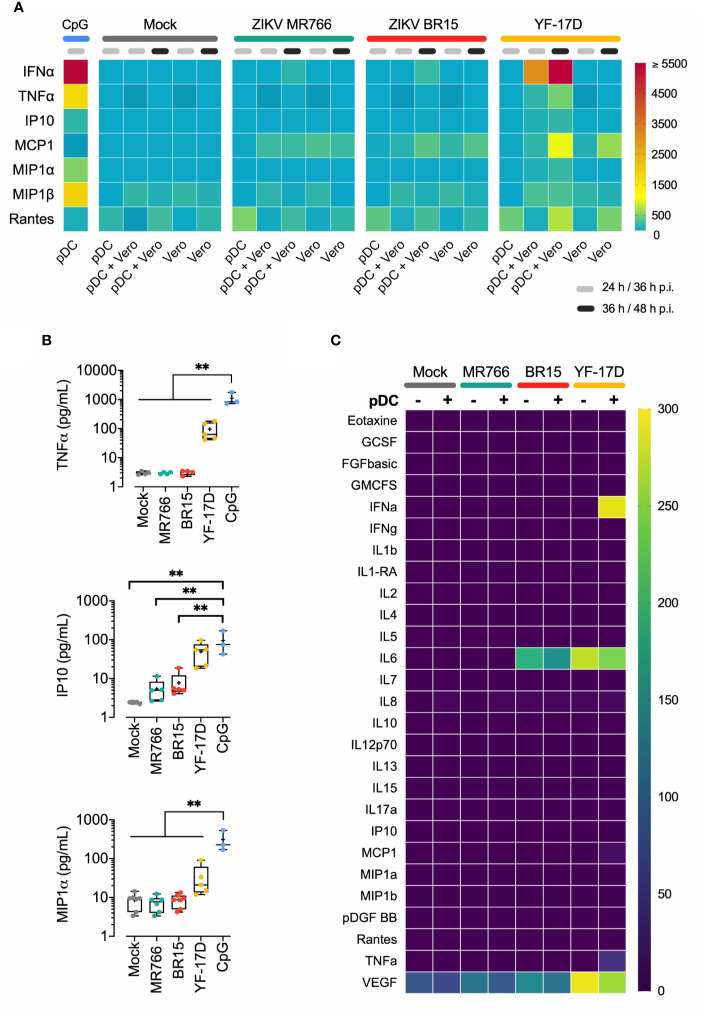
No inflammatory response in pDCs exposed to ZIKV-infected cells **(A)** Quantification of cytokines and chemokines in the supernatant of pDCs stimulated with ODN 2216, pDCs exposed to indicated cell free virus (MOI of 1) tested at 24 h, or pDCs cocultured with Vero cells infected with both strain of ZIKV or YF-17D (MOI of 1) and tested 36 and 48 h p.i. Heat map was used to visualize the broad array of cytokines and chemokines produced by pDCs upon ZIKVs and YF-17D exposure. The colored scale bar shows the range of concentration expressed in pg/ml. Concentrations shown are those from one representative donor. **(B)** Concentrations of TNFα, IP10, and MIP1α produced by pDCs mock infected, co-cultured with Vero cells infected with the indicated flaviviruses at MOI of 1, or stimulated with ODN 2216 for 24 h. Mean/SD of three (ODN) to five (cocultures) distinct donors. **(C)** Heatmap of cytokines and chemokines released by pDCs either mock infected, or cocultured with IMR-32 cells infected with the indicated flaviviruses at MOI of 1 and tested 36 h post-infection. The pattern of immune mediators released by infected IMR-32 cells alone is shown (mean of two donors). **p < 0.001.

pDCs are critical in bridging innate and adaptive immune responses in the context of systemic viral infections, in part by the production of IFN-α, but also through their role in establishing an inflammatory microenvironment characterized by broad array of cytokines/chemokines. Specifically, TLR-7 or -9 agonists trigger a proinflammatory response in pDCs, characterized by four distinct cytokine loops in respect to the pDCs ability to produce type I IFNs (29). In the first loop, activated pDCs secrete mediators such as TNFα and MIP-1α in response to TLR-engagement, but independently of IFN-α/ß receptor (IFNAR) stimulation. In the second loop, IL-8 is the only molecule whose production by pDCs is inhibited by IFNAR signaling. In the third loop the release of IP-10 and MIP-1ß is enhanced by autocrine IFN-α**/**ß, while cytokines in the fourth loop, such as MCP1, are not directly produced by pDCs but by other cell types in the microenvironment under the influence of IFN-α**/**ß (29). Taking advantage of multianalyte profiling (MAP) technology, we have performed an in-depth analysis of the cytokines and chemokines secreted by pDCs activated by CpG ODN 2216, and compared their expression pattern in response to cell free or cell-associated ZIKV MR766 or BR15. In these experiments, YF-17D served as a positive control. The pattern of molecules produced by Vero cells infected with corresponding viruses and the one of mock infected cells was also determined. A heat map of the immune mediators detected under these various conditions is shown in **Figure 5A**. This MAP analysis reveals that no inflammatory cytokines and chemokines were produced by pDCs in response to ZIKV MR766 or BR15, in contrast to pDCs triggered by YF-17D-infected Vero cells. **Figure 5B** compares mean concentrations of TNF-α, IP-10, and MIP-1α secreted by pDCs in response to either ODN 2216, YFV, or ZIKVs from experiments performed with pDCs sorted from 6 donors.

We also assessed the inflammatory response of pDCs exposed to virus-infected IMR-32 cells. The heat map, depicted in **Figure 5C**, shows that, regardless the virus, no inflammatory response was induced in pDCs. Constitutive low production of VEGF by IMR-32 cells was detected and was strongly increased by YF-17D infection. No IFN-α response was induced in pDCs triggered by MR766 or BR15 ZIKV, confirming the data in **Figure 4C**. IL-6 detected in the SN of IMR-32 cells infected by YF-17D or ZIKV BR15 is exogenous and comes from the viral inoculum produced on Vero cells, which are strong producers of IL-6 (30, 31) (**Figure S4**). Overall, these data clearly show that, in contrast to YF-17D, both ZIKV strains were unable to trigger an inflammatory response in pDCs.

### ZIKV NS1 Induces Elevated SYK Phosphorylation

**Figure 6:**
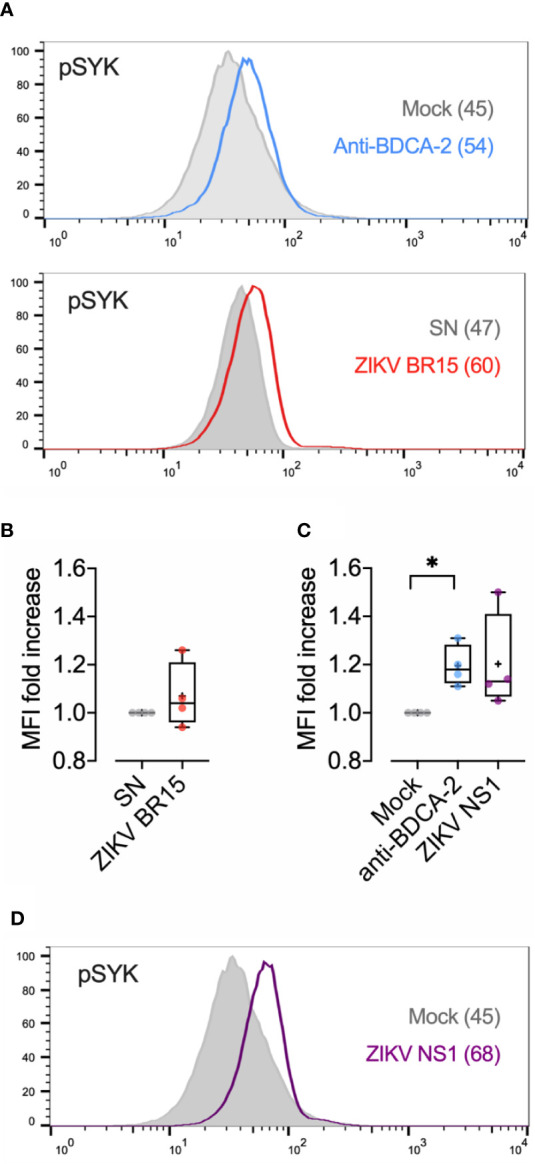
ZIKV Induces SYK phosphorylation **(A):** Purified pDCs were incubated with 120 µl of complete medium (Mock) or ZIKV BR15 (equivalent MOI of 3) for 30 min. Positive control with anti-BDCA-2 antibody (2 μg/ml) for 30 min is shown. Cells were harvested, permeabilized and intracellularly stained for pSYK (Y525/526) and analyzed by flowcytometry, as described in the *Materials and Methods*. Shown are representative histograms from independent experiments performed with pDCs from three donors. **(B)** pDCs were incubated for 30 min with virus free SN or BR15 at MOI of 5. The percentage of pDCs expressing intracellular pSYK Y525/526 was determined. Mean fluorescence intensity (MFI) was also determined. The experiment was performed with sorted pDCs from 4 donors and mean/SD results are shown. **(C)** Purified pDCs were incubated in PBS/BSA 2% (mock) or incubated for 30 min with anti-BDCA-2 antibody (2 μg/ml) or ZIKV NS1 (5 μg/ml). The percentage of pDCs expressing intracellular pSYK Y525/526 and MFI were determined. The experiment was performed with sorted pDCs from four donors and mean/SD results are shown. The percentage of pDCs upregulating pSYK following incubation with NS1 was statistically significant as compared to mock (p < 0.01), as well as MFI fold increase. **(D)** Histogram of pDCs incubated 30 min with NS1 (5 μg/ml) or medium. MFI is indicated in brackets. *p < 0.05.

CD303 (BDCA-2) has been identified as an inhibitory receptor on pDCs. CD303 cross-linking induces a BCR-like signaling that significantly limits both IFN-α production and NF-κB pathway activation, and impairs pDCs maturation (17, 32–34). Given that pDCs exposed to ZIKV displayed a behavior stamped by the same features, we investigated whether this unexpected lack of anti-viral response was caused by CD303 triggering. Stimulation of CD303 is known to rapidly induce the transient phosphorylation of the spleen tyrosine kinase (SYK) at tyrosine residue 352 and, more distinctly, at residue Y525/526 (35). Using antibody specific to phosphorylated SYK Y525/526, we performed a Phosflow assay to evaluate intracellular phosphorylated SYK (pSYK) in pDCs incubated with either the epidemic ZIKV strain (BR15) or its matched virus-free inoculum (SN). Representative histograms from three independent experiments are shown in **Figure 6A**.

The upregulation of intracellular pSYK was detected within 30 min of exposure of pDCs to ZIKV BR15, suggesting that ZIKV is capable of signaling through CD303. This upregulation was at levels comparable to pDCs stimulated with cross-linking anti-CD303 (BDCA-2) antibody. The mean percentage of pDCs staining positive for pSYK Y525/526 and mean fluorescence intensity (MFI) 30 min after BR15 exposure of pDCs from three donors are shown in **Figure 6B**. We then searched for a potential CD303 ligand common to MR766 and BR15. Given that CD303 is a type II C-type lectin receptor, we sought a potential ligand among the glycosylated proteins of ZIKV. Since both ZIKV MR766 and BR15 inhibited the interferon response of pDCs, the envelope protein was excluded given that only the E of BR15 is glycosylated. In contrast, both ZIKV strains encode a non-structural protein 1 (NS1) with two N-linked glycosylation sites (N130 and N207) (36) which, due to its secretion in the extracellular medium was contained in viral inoculum. pDCs were thus left mock-stimulated or stimulated with pure ZIKV NS1 or anti-CD303 (BDCA-2) antibody for 30 min. **Figures 6C, D** show that NS1 induced SYK phosphorylation at residue Y525/526, as observed on the histogram. Mean percentage of pDCs staining positive for pSYK Y525/526 and mean MFI after 30 min incubation with NS1 of pDCs from three donors were comparable to that of pDCs triggered with anti-CD303 (BDCA-2) antibody, and significantly higher than pDCs in mock cultures (**Figure 6D**). These results demonstrate that both molecules induced the rapid phosphorylation of SYK at residue Y525/526, thereby revealing the unexpected potency of ZIKV NS1 to activate the inhibitory CD303 signaling cascade, thus resulting in the inhibition of pDCs maturation and IFN I response.

## Discussion

Type I IFN response plays a critical role in the control of flaviviruses, as shown by the increased susceptibility of mice lacking components of the IFN pathway to flavivirus infection (7, 37) and the numerous mechanisms employed by flaviviruses to counteract this control (38, 39). Here, we report that human sorted pDCs are not susceptible to infection by cell-free ZIKV virions, while they can be infected when exposed to ZIKV-infected cells, as evidenced by intracellular dsRNA expression and viral protein detection by confocal microscopy. However, pDCs were unable to mount an IFN-α response, whether exposed to simian infected-epithelial cells or human infected-neuroblastoma cells. In addition, no inflammatory response was detected, and they poorly matured when exposed to ZIKV. This defective innate antiviral response was accompanied by SYK phosphorylation following the engagement of CD303 by ZIKV NS1.

pDCs are not permissive to most viral infections and recent studies exploring pDC activation by flaviviruses have revealed that sensing of virus-infected cells by pDCs was more effective than sensing of circulating cell-free viruses. This requirement for cell-cell contact is increasingly recognized as a hallmark of pDC-mediated antiviral state, triggered by evolutionarily divergent enveloped RNA viruses (40). This was reported for several arboviruses, including DENV (14, 40), WNV (14), YFV (15), and CHIKV (40). In this study, we report that primary human pDCs are refractory to cell-free ZIKV too. Nevertheless, cell-cell contact between pDCs and Vero cells infected with both historical or epidemic ZIKV strains led to the infection of a small fraction of pDCs, detected by intracellular expression of viral dsRNA, and confirmed their permissiveness through indirect route of infection. Our observations are consistent with a recent study assessing the frequency of ZIKV-infected cells in circulating blood cell populations from individuals with naturally acquired acute infection. Indeed, Sun et al. (13) reported that ZIKV RNA was mainly detected in sorted mDCs whereas pDCs, B cells, NK cells, CD4, and CD8 T cells of the patients tested contained no detectable viral RNA. This was corroborated by *in vitro* infection experiments on PBMCs since highest levels of ZIKV transcripts were observed in mDCs and lower levels of ZIKV transcripts were detected in the other subsets, including pDCs (41).

Several features characterize the antiviral state of pDCs, such as a robust production of IFNα, concomitant with additional antiviral responses, including inflammatory cytokine secretion. While the lack of cytokine response was expected in pDCs exposed to cell-free virions (15, 42), it was striking to observe that pDCs co-cultured with ZIKV-infected cells triggered a barely detectable IFN-α response and no production of inflammatory mediators. This surprising observation is in sharp contrast to previous studies conducted with related flaviviruses such as YFV (43), DENV (14), and WNV (42) in which co-culture experiments induced a robust IFN response. It is also in contrast to the study from Assil et al. (44) who reported robust pDC IFNα productions induced when pDCs were in physical contact with ZIKV-infected cells. Nevertheless, our results are consistent with a recent report assessing ZIKV-induced IFN response in human PBMC, which showed the complete lack of type I and type III IFN induction by ZIKV, suggesting the ability of ZIKV to evade the type I IFN signaling pathway (45). In addition, a remarkable downregulation of antiviral IFN-stimulated genes and innate immune sensors in mDCs was reported (41), suggesting that ZIKV can actively suppress Interferon-dependent immune responses. Moreover, transcriptional profiling of whole blood cells isolated from patients with acute infection by ZIKV indicated that ISG and innate immune sensors remained inactive as compared to those of healthy donors, suggesting that type I IFN response is suppressed during the natural infection process in humans (46). The importance of type I IFN in mediating host restriction of ZIKV is evident through studies in murine models, which have shown that mice producing very low amount of IFNα/ß or lacking the IFN receptor are highly susceptible to ZIKV-induced illness (7). Indeed, a genetic deficiency in type I IFN signaling shifts the balance to sustained viral replication and disseminated disease, promoting spread to the CNS and lethal infection (7, 47). ZIKV has developed several strategies to antagonize type I IFN signaling to evade the pressure of host innate immune responses. Bowen et al. (16) recently reported that human myeloid DCs, targets of mosquito-borne flaviviruses, are susceptible to productive viral replication, yet these cells failed to secrete type I or III IFN. The defect was due to a selective inhibition of translation of type I IFN proteins, while translation of other antiviral host proteins remained intact. ZIKV non-structural proteins, NS1, NS4A, and NS5, may also inhibit type I IFN through inhibition of IRF3 and NF-κB signaling (48). In addition, ZIKV is also able to counteract type I IFN responses by blocking phosphorylation of STAT1 and STAT2, thus antagonizing JAK/STAT signaling (16). Our study reveals a new mechanism exploited by ZIKV to block IFN response in pDCs, and the unsuspected ability of ZIKV NS1 to trigger the CD303 signaling. Thus, ZIKV targets multiple points within the type I IFN induction signaling cascade. Altogether, these studies illustrate the remarkable ability of ZIKV to evade the pressures of host innate immune responses.

Upon sensing of viral pathogen, pDCs undergo maturation with coordinated regulation of surface markers that mediate important pDCs functions such as interaction with other immune cells or migration to secondary lymphoid tissues. While exposure to ZIKV did not trigger an increase in the surface expression of CD83, CD86, and HLA-DR maturation markers in pDCs, a slight upregulation of CCR7 and a downregulation of CD303 were observed. CD303 is a C-type lectin expressed in large amount on the surface of human pDCs (34). CD303 engagement leads to its rapid internalization and reduced levels of transcripts for IFN-I genes and IFN-I-responsive genes (17, 34). In addition, CD303 signaling cascade is a putative regulator of the canonical NF-κB pathway activity, and thereby subsequent inflammatory cytokine gene expression (17). Therefore, we sought to determine whether the defect in pDC maturation, in particular the inhibition of CD86 up-regulation reported to be triggered by CD303 signaling (26), as well as the inhibition of IFN-α production and minimal inflammatory cytokine secretion when pDCs were exposed to ZIKV, could be a result of CD303 signaling. CD303 is an endocytic C-type lectin receptor able to bind and internalize glycosylated antigens that induces a BCR-like signaling cascade involving SYK phosphorylation (17). ZIKV can encode up to three glycosylated proteins depending on the viral strain, including the non-structural protein 1 (NS1) and two structural proteins, the envelope (E) and the membrane precursor (prM). The Envelope protein was excluded given that ZIKV MR766 particles do not harbor a glycosylated envelope protein on the virion surface (49, 50). Nonstructural ZIKV proteins were shown to cooperate *in vitro* to help the virus evade interferon-mediated antiviral response (51). In particular, NS1, a multifaceted protein well known for its critical role in flavivirus biology and pathogenesis, inhibits the IFN-β signaling pathway by targeting TBK1 phosphorylation (51, 52). In addition, NS1 is the only Flavivirus protein secreted from the cells and circulates at significant levels in the plasma of infected people (53). Our study proposes a new mechanism of ZIKV immune evasion through NS1-mediated CD303 signaling that can inhibit IFN-α expression by pDCs, thus antagonizing the innate IFN type-I antiviral response. CD303-mediated inhibition of IFN-α production has been reported for other viral proteins, such as the hepatitis B surface antigen (HbsAg) (54), HIV gp120 (55), and HCV glycoprotein E2 (56). Thus, this property is shared by viruses belonging to three different viral families, suggesting that this antiviral inhibitory pathway is an important immune evasion strategy in a variety of viral infections. Specific treatments and vaccines for ZIKV are not currently available. Our study identifies NS1 as a potential therapeutic target to restore the sentinel immune function of pDCs. Human antibodies specific for NS1, isolated from an individual with symptomatic ZIKV infection, were found to confer a Fc-dependent protection against disease and death after a challenge with African and Asian lineage strains of ZIKV in Stat2–/– mice, thus demonstrating the protective role of mAbs targeting the NS1 protein (57). Active protective immunogenicity of NS1 was also demonstrated with NS1-based vaccine candidates which elicited protective high antibody titers (58), or protection from systemic ZIKV infection by NS1-specific T cell responses in immunocompromised mice (59). However, the protective role of anti-NS1 antibodies in mice must be independent of CD303 triggering and SYK phosphorylation since this receptor is not expressed in mice.

The mechanism by which ZIKV enters the central nervous system through the peripheral entry route remains to be determined. However, it is likely that the virus gains access to target neural tissue either as cell-free virus, through disruption of the blood-brain barrier for example, or through infected immune cells, able to circumvent this latter. This possibility is supported by the fact that ZIKV can infect pDCs by cell-to-cell contact. Such immunocompetent cells are present in the skin, the main entry route of ZIKV infection, but they are also able to infiltrate the CNS, as reported in the setting of autoimmune diseases (60). Through ZIKV-induced expression of CCR7, a receptor involved in pDC trafficking involving its interaction with the ligands CCL19 and CCL2127, infected pDCs could migrate from primary infected to secondary lymphoid tissues (25)). Interestingly, CCL19 and CCL21 are constitutively expressed in the CNS (61). Therefore, it is conceivable that infected pDCs serve as a Trojan horse for ZIKV entry into the CNS by taking advantage of the ability of specific blood-brain barrier components to attract CCR7-expressing cells.

## Data Availability Statement

The raw data supporting the conclusions of this article will be made available by the authors, without undue reservation.

## Author Contributions

SB, PD, GG, and M-LG conceived and planned the study. SB, BP-B, VS, CM, and MM carried out the experiments. SB, BP-B, MM, PD, and GG analyzed and interpreted the data. M-LG supervised the project and took the lead in writing the manuscript. All authors contributed to the article and approved the submitted version.

## Funding

This work was supported by Institut Pasteur and the POE FEDER 2014-20 of the Conseil Régional de La Reunion (ZIKAlert program, N° SYNERGIE RE00001902) and by the INCEPTION project (PIA/ANR-16-CONV-0005). SB was supported by a PhD grant from MEESR (Ecole Doctorale STS, Université de La Réunion).

## Conflict of Interest

The authors declare that the research was conducted in the absence of any commercial or financial relationships that could be construed as a potential conflict of interest.

## Supplementary Material

Supplementary Figure 1 Freshly sorted pDCs (Fresh) were either incubated with medium (Mock) or virus-free supernatant (SN), or exposed to indicated ZIKV strain at MOI 1 or 5 for 24 h and the frequency of apoptotic cells among CD123^+^ cells was determined with 7-AAD dye. Representative dot plots are shown on the left panel, and the mean frequency of apoptotic pDCs (CD123^+^7-AAD^+^) under the various conditions is shown on the right panel. The error bars represent the standard deviations of two experiments conducted with primary cells from two distinct donors.

Supplementary Figure 2 IFN-α production by sorted pDCs exposed to cell free ZIKV. IFN-α production by sorted pDCs exposed 24 h to cell free virions from MR766 or BR15 ZIKV strain at MOI of 5 or YF-17D at MOI of 1 or incubated in medium. pDCs stimulated by ODN 2216 is the positive control. Mean/SD of results from three experiments performed on pDCs sorted from three distinct donors.

Supplementary Figure 3 Susceptibility of Vero cells to ZIKV infection in the presence of pDCs. Vero cells were left uninfected (mock) or infected with the indicated flavivirus strain at MOI of 1 for 36 and 48 h in the absence or presence of pDCs. **(A)** Percentage of infected Vero cells, as assessed by intracellular staining with J2 anti-dsRNA antibody at 36 h post infection. Mean/SD of results obtained from two experiments performed in triplicate. **(B)** Quantification of viral progeny production. **(C)** Analysis of cell viability. Virus-induced cell death was assessed by LDH release measurement. Cell viability is expressed as percentage relative to maximum LDH release. Mean/SD results from three experiments in panels **(B)** and **(C).**

Supplementary Figure 4 Cytokine/chemokine pattern of Vero cells exposed to ZIKV. Quantification of immune mediators in the supernatant of Vero cells exposed to both strains of ZIKV or YF-17D (MOI of 1), tested at 36 and 48 h p.i. Heat map was used to visualize the broad array of cytokines and chemokines produced. The colored scale bar shows the range of concentration expressed in picogram per milliliter (pg/ml). Concentrations shown are those from one representative experiment.
