# Supplementary figures and images for: Zika Virus Inhibits IFN-α Response by Human Plasmacytoid Dendritic Cells and Induces NS1-Dependent Triggering of CD303 (BDCA-2) Signaling
Source: Front Immunol. 2020 Oct 28;11:582061. doi: 10.3389/fimmu.2020.582061 (PMC7655658; doi:10.3389/fimmu.2020.582061)

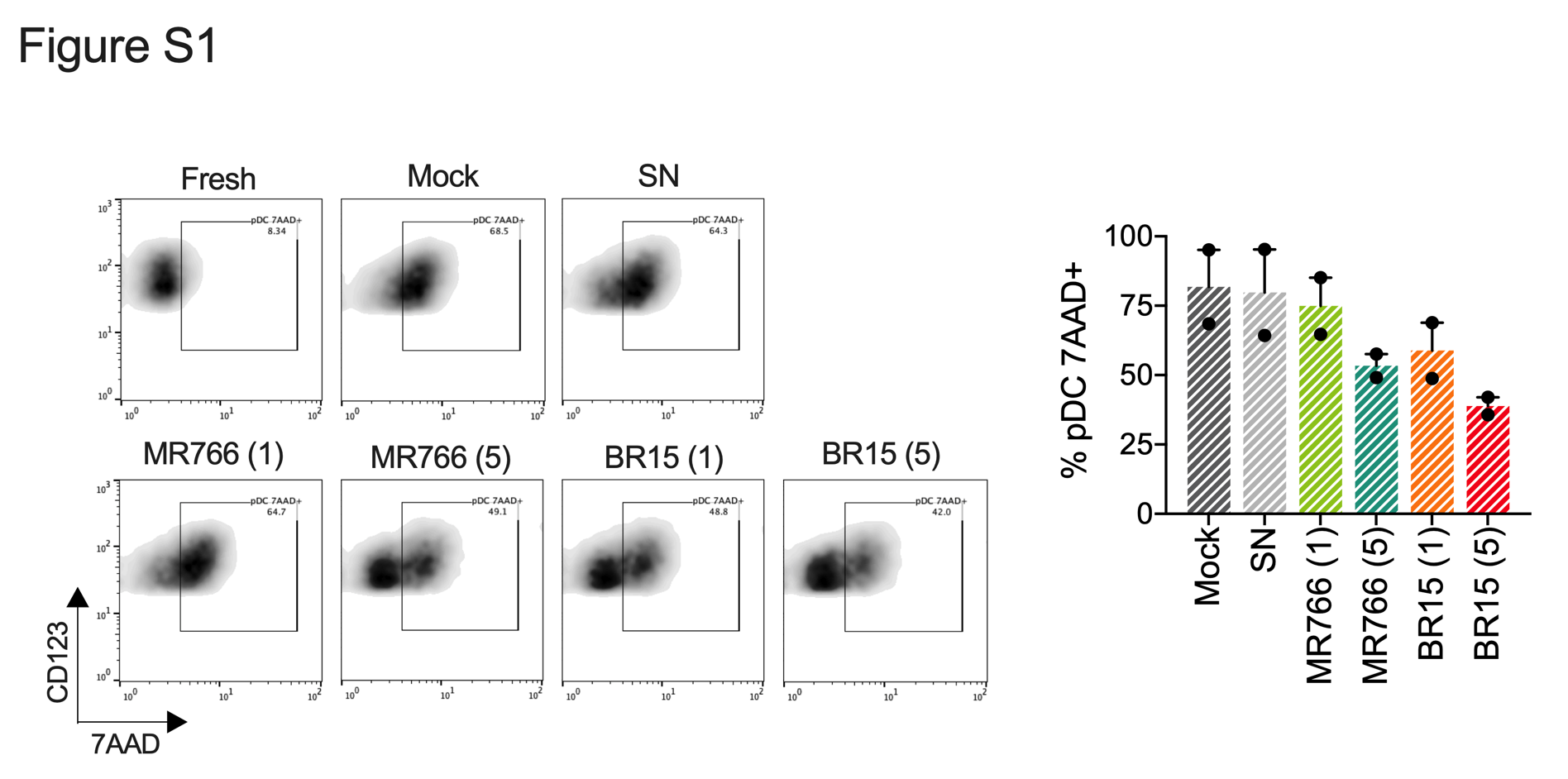

Supplement: Supplementary Figure 1 — Freshly sorted pDCs (Fresh) were either incubated with medium (Mock) or virus-free supernatant (SN), or exposed to indicated ZIKV strain at MOI 1 or 5 for 24 h and the frequency of apoptotic cells among CD123+ cells was determined with 7-AAD dye. Representative dot plots are shown on the left panel, and the mean frequency of apoptotic pDCs (CD123+7-AAD+) under the various conditions is shown on the right panel. The error bars represent the standard deviations of two experiments conducted with primary cells from two distinct donors. [file Image_1.tiff]

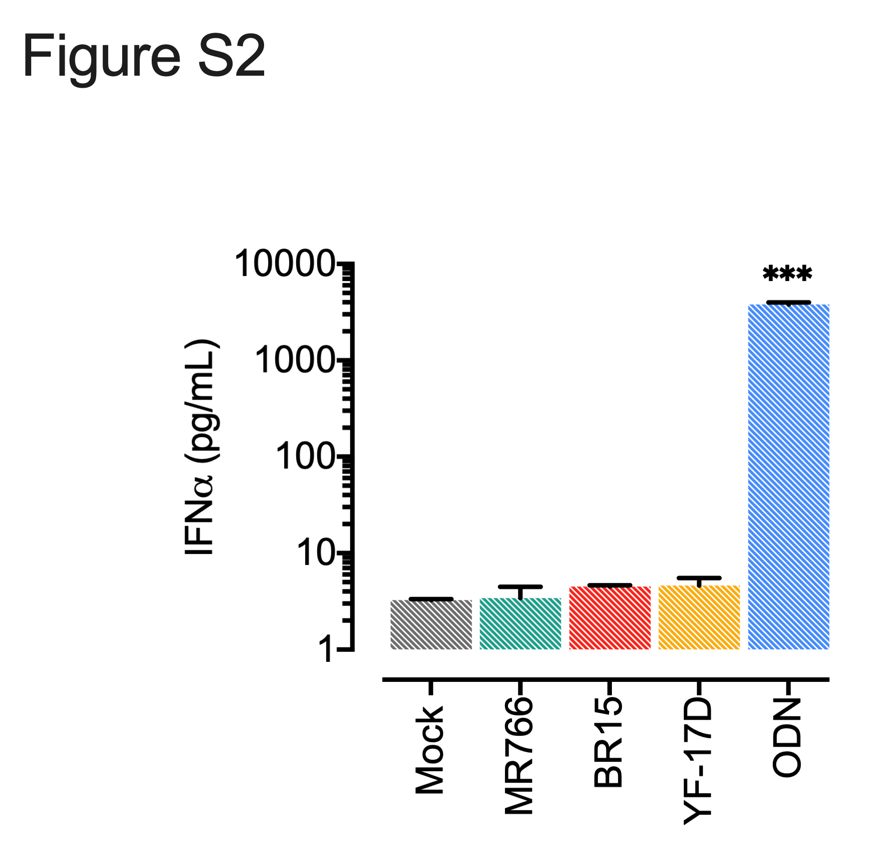

Supplement: Supplementary Figure 2 — IFN-α production by sorted pDCs exposed to cell free ZIKV. IFN-α production by sorted pDCs exposed 24 h to cell free virions from MR766 or BR15 ZIKV strain at MOI of 5 or YF-17D at MOI of 1 or incubated in medium. pDCs stimulated by ODN 2216 is the positive control. Mean/SD of results from three experiments performed on pDCs sorted from three distinct donors. [file Image_2.tiff]

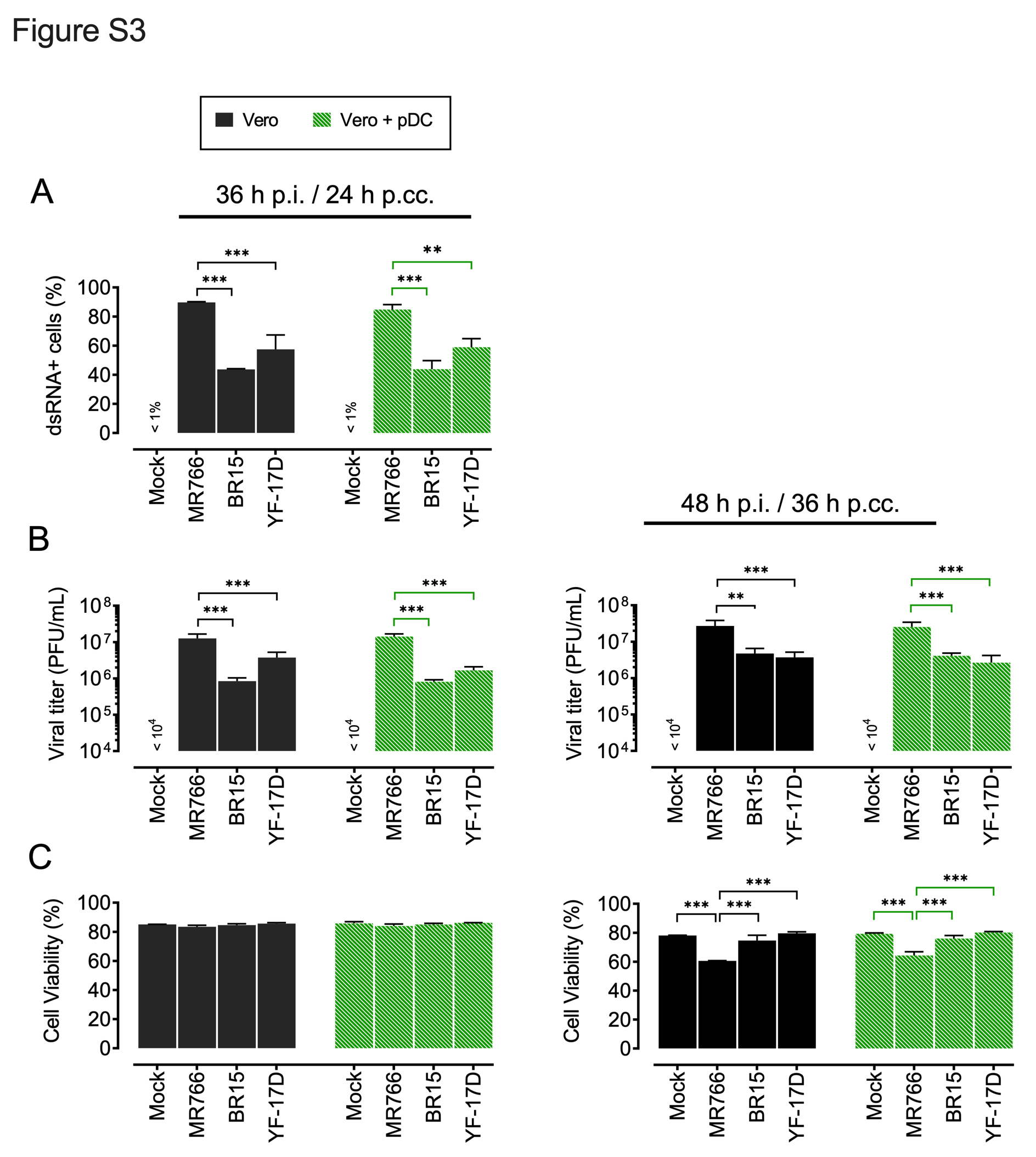

Supplement: Supplementary Figure 3 — Susceptibility of Vero cells to ZIKV infection in the presence of pDCs. Vero cells were left uninfected (mock) or infected with the indicated flavivirus strain at MOI of 1 for 36 and 48 h in the absence or presence of pDCs. (A) Percentage of infected Vero cells, as assessed by intracellular staining with J2 anti-dsRNA antibody at 36 h post infection. Mean/SD of results obtained from two experiments performed in triplicate. (B) Quantification of viral progeny production. (C) Analysis of cell viability. Virus-induced cell death was assessed by LDH release measurement. Cell viability is expressed as percentage relative to maximum LDH release. Mean/SD results from three experiments in panels (B) and (C). [file Image_3.tiff]

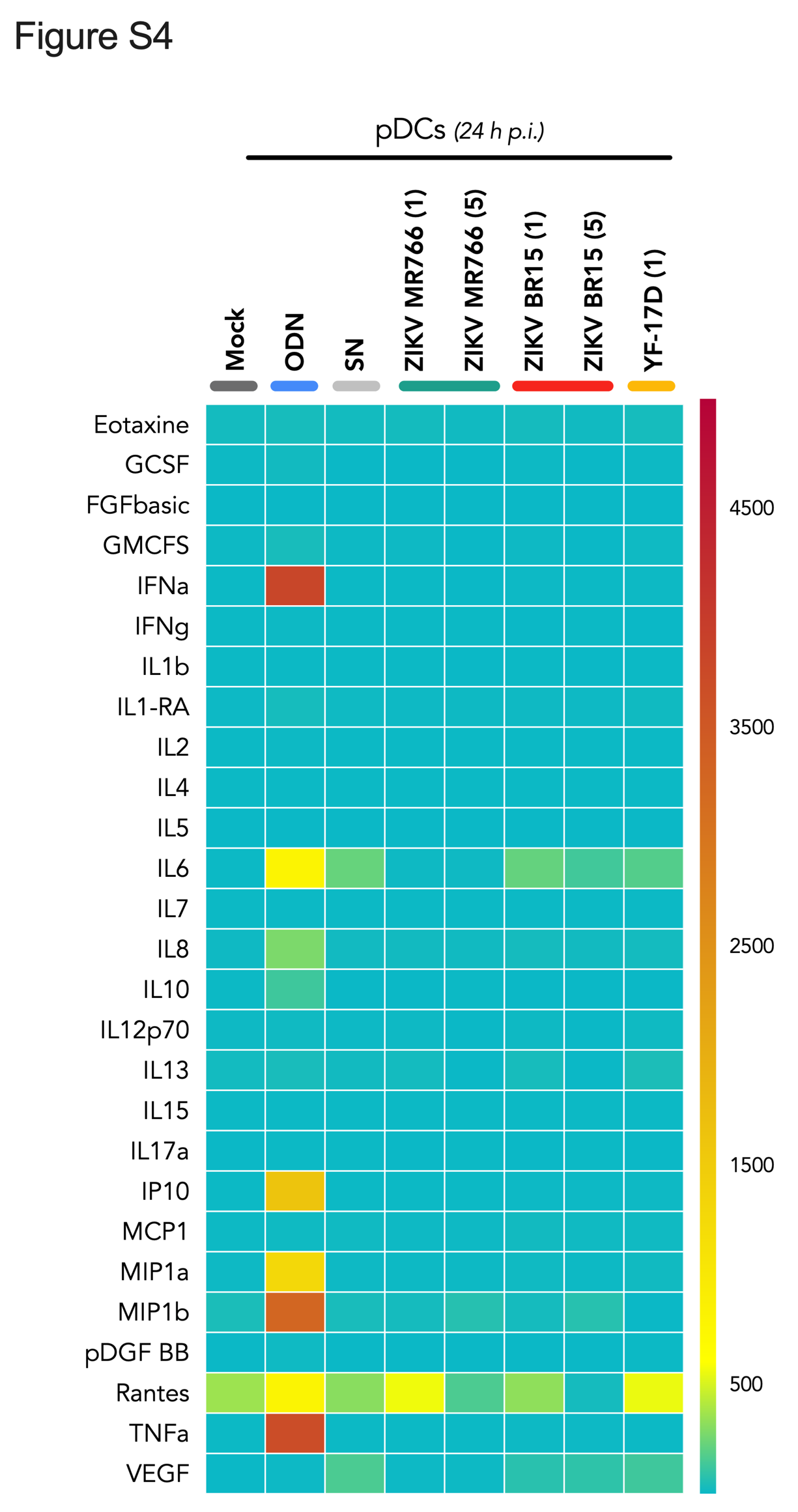

Supplement: Supplementary Figure 4 — Cytokine/chemokine pattern of Vero cells exposed to ZIKV. Quantification of immune mediators in the supernatant of Vero cells exposed to both strains of ZIKV or YF-17D (MOI of 1), tested at 36 and 48 h p.i. Heat map was used to visualize the broad array of cytokines and chemokines produced. The colored scale bar shows the range of concentration expressed in picogram per milliliter (pg/ml). Concentrations shown are those from one representative experiment. [file Image_4.tiff]
