# Supplementary material for: Zika Virus Inhibits IFN-α Response by Human Plasmacytoid Dendritic Cells and Induces NS1-Dependent Triggering of CD303 (BDCA-2) Signaling
Source: Front Immunol. 2020 Oct 28;11:582061. doi: 10.3389/fimmu.2020.582061 (PMC7655658; doi:10.3389/fimmu.2020.582061)
Supplement: Supplementary file 5 [file Table_1.docx]

Supplemental Table 1

**Adjusted p.value of Fig 1C representing pDCs frequency in SSC^low^ cells (ANOVA, Holm-Sidak's multiple comparisons test)**

|  | MR766 (1) | MR766 (5) | BR15 (1) | BR15 (5) | Mock | SN | CpG | Fresh |
| --- | --- | --- | --- | --- | --- | --- | --- | --- |
| MR766 (1) | - | *** | * | *** | ns | ns | ** | *** |
| MR766 (5) | 0,0004 | - | ns | ns | *** | * | ns | *** |
| BR15 (1) | 0.0176 | 0,3412 | - | ** | *** | ns | ns | *** |
| BR15 (5) | <0.0001 | 0,3412 | 0.0024 | - | *** | *** | ns | *** |
| Mock | 0.4815 | <0.0001 | 0.0008 | <0.0001 | - | * | *** | *** |
| SN | 0.3412 | 0.0220 | 0,3916 | <0.0001 | 0.0434 | - | ns | *** |
| CpG | 0.0011 | 0.7082 | 0.3916 | 0.1876 | <0.0001 | 0,0549 | - | *** |
| Fresh | <0.0001 | <0.0001 | <0.0001 | <0.0001 | <0.0001 | <0.0001 | <0.0001 | - |
